# A problem with variable selection in a comparison of correlative and process‐based species distribution models: Comments on Higgins et al., 2020

**DOI:** 10.1002/ece3.7496

**Published:** 2021-09-06

**Authors:** Trevor H. Booth

**Affiliations:** ^1^ CSIRO Land and Water Canberra Australian Capital Territory Australia

**Keywords:** Acacia, BIOCLIM, ecological niche models, eucalypt, invasive species, species distribution models

## Abstract

Comments are presented on an article published in October 2020 in *Ecology and Evolution* (“Predictive ability of a process‐based versus a correlative species distribution model”) by Higgins et al. This analyzed natural distributions of Australian eucalypt and acacia species and assessed the adventive range of selected species outside Australia. Unfortunately, inappropriate variables were used with the MaxEnt species distribution model outside Australia, so that large climatically suitable areas in the Northern Hemisphere were not identified. Examples from a previous analysis and from the use of the freely available spatial portal of the Atlas of Living Australia are provided to illustrate how the problem can be overcome. The comparison of methods described in the Higgins et al. paper is worthwhile, and it is hoped that the authors will be able to repeat their analyses using appropriate variables with the correlative model.

## Graphical abstract

The Higgins et al., (2020) MaxEnt species distribution modeling analysis used inappropriate variables outside Australia. As a result, large climatically suitable areas for Acacia saligna in the Northern Hemisphere, especially in the Mediterranean Basin, were not identified

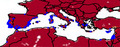

The analysis of suitable environmental conditions for Australian tree species, both in Australia and overseas, has been a recurrent theme since the first species distribution model (SDM) package became available in 1984 (see sections 2.2 and 3.0 in Booth & Muir, 2020). The Higgins et al., (2020) paper analyzed natural distribution data for 664 Australian eucalypt and acacia species. Natural distribution data for 46 selected species were also analyzed to assess whether a process‐based or a correlative SDM most effectively predicted the adventive range of these species outside Australia. It was concluded that “the correlative model—MaxEnt—has a superior ability to describe the data in the training data domain (Australia) and that the process‐based model—TTR‐SDM—has a superior ability to predict the distribution of the study species outside of Australia.” These conclusions may be true, but readers cannot be sure as there is a major problem with the variables selected for use when applying the correlative model outside Australia. This causes the MaxEnt model (Phillips et al., 2006) to fail to identify large environmentally suitable areas in the Northern Hemisphere.

In their methods section, Higgins et al., (2020) described that their process‐based modeling used monthly data for minimum, mean and maximum monthly temperature, soil moisture contents, and solar radiation. A soil nitrogen content measure was also used that was held constant across all months. They stated that “This exact same list of environmental variables was used for the MaxEnt model fitting. This ensures that both models use the same information for estimating the species distribution models.”

The problem with using monthly variables in a correlative model for the whole world is that the same months are not comparable across hemispheres. To avoid this problem, climatic classifications, such as the widely used Köppen and Köppen–Geiger systems (Köppen, 1936; Peel et al., 2007), were developed using measures related to periods such as seasons (i.e., summer or winter) and the coldest month. Early SDM studies built on these ideas but provided sets of variables rather than a hierarchical classification system. The first SDM package was called BIOCLIM (Booth et al., 2014; Nix, 1986) and used a set of 12 variables, which was extended to 19 variables in 1996. These variables are listed in the ANUCLIM publication (for current version, see Xu & Hutchinson, 2011), which describes several programs including BIOCLIM. The set of 19 BIOCLIM variables was adopted for use by the WorldClim system (Fick & Hijmans, 2017; Hijmans et al., 2005), which with over 20 000 citations in Google Scholar^TM^ is the most widely used source of climatic data for SDM studies.

Higgins et al., (2020) provided an example of their comparative analyses in their Figure 5. The top map in this figure shows “global projected environmental suitability” based on a MaxEnt analysis of the natural distribution of *Acacia saligna* in Western Australia. Only very limited areas of climatic suitability are shown in Figure 5 in the Northern Hemisphere. These are mainly in the Middle East, particularly in southwestern Iran and northern Iraq. Their figure shows that there are no occurrence records for *A. saligna* available from the Global Biodiversity Information Facility (GBIF) in these areas.

Some of the main areas in which *A. saligna* is found naturally in Australia are in Csa and Csb regions of the Köppen–Geiger climate classification, that is, hot‐warm summer/Mediterranean (winter rainfall) climates. Not surprisingly, there are extensive Csa and Csb regions in the actual Mediterranean region, as well as in California as shown by the maps of Peel et al., (2007). In contrast, the Higgins et al., (2020) MaxEnt analysis indicates almost no climatically suitable areas in the Mediterranean region and none in California, though blue crosses indicate numerous occurrences of *A. saligna* in the Mediterranean (see Figure 1) and some in California as recorded by the GBIF.

**FIGURE 1:**
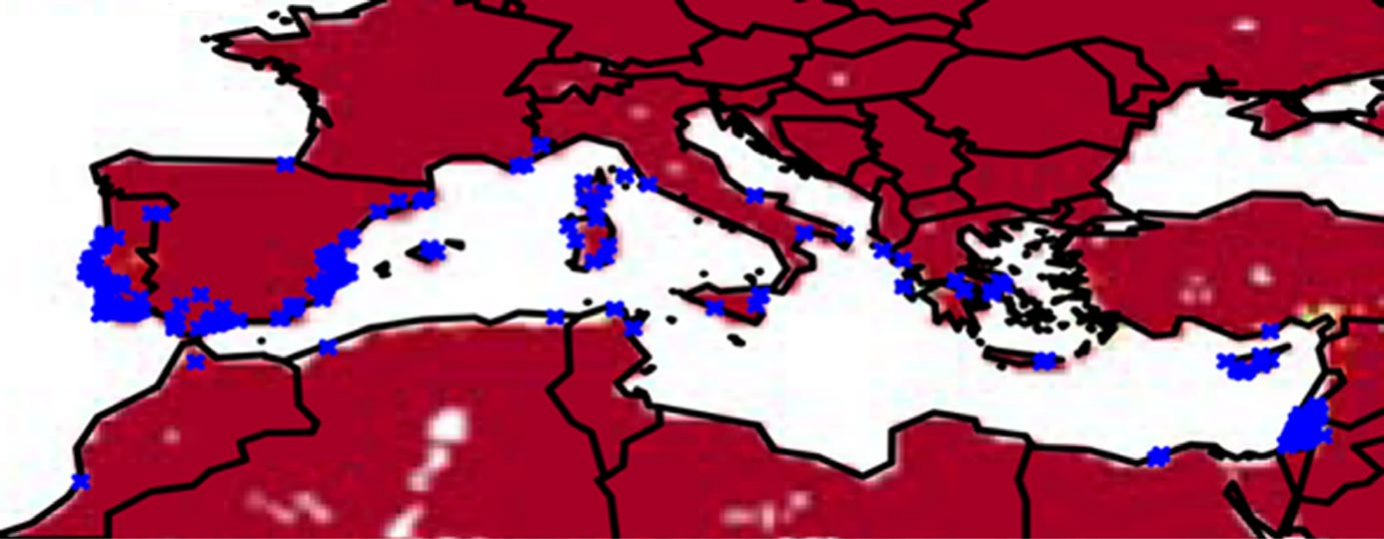
Environmental suitability of *A. saligna* for the Mediterranean region based on a MaxEnt analysis of the natural distribution by Higgins et al., (2020). Dark red indicates an environmental suitability of 0. Blue crosses indicate occurrences of *A. saligna* obtained from the Global Biodiversity Information Facility (GBIF)

The difference between the Higgins et al., (2020) MaxEnt analysis for the Mediterranean region and what might be expected can be seen even more clearly when it is compared with a MaxEnt analysis provided by Thompson et al., (2011) of the Mediterranean region (see Figure 2). This was also based on an analysis of the natural distribution of *A. saligna* in Western Australia. However, Thompson et al., (2011) used variables developed for the BIOCLIM model. These were the following: temperature seasonality (Bio4), mean temperature of the hottest quarter (Bio10), mean temperature of the coldest quarter (Bio11), precipitation seasonality (Bio15), precipitation of the hottest quarter (Bio18), and precipitation of the coldest quarter (Bio19).

**FIGURE 2:**
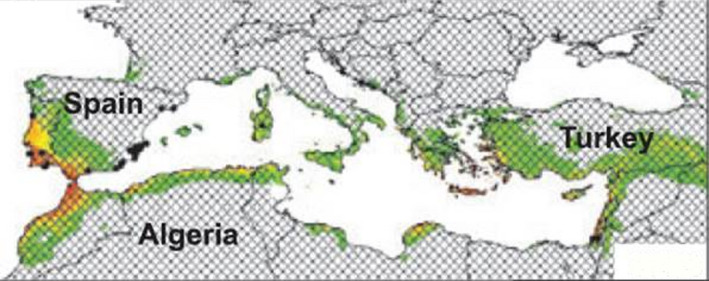
Environmental suitability of *A. saligna* for the Mediterranean region based on a MaxEnt analysis of the natural distribution by Thompson et al., (2011). Green‐yellow areas are climatically suitable and reddish‐brown areas are highly suitable. Note the extensive suitable areas compared with the almost total lack of suitable areas in Figure 1

Using the spatial portal of the Atlas of Living Australia (ALA, https://www.ala.org.au/, Belbin, 2011), a MaxEnt analysis for *A. saligna* was carried out for the whole world using the six BIOCLIM variables employed by Thompson et al., (2011) (see Figure 3). In contrast to Figure 5 of Higgins et al., it shows extensive areas of climatic suitability around the Mediterranean and limited climatically suitable areas in California.

**FIGURE 3:**
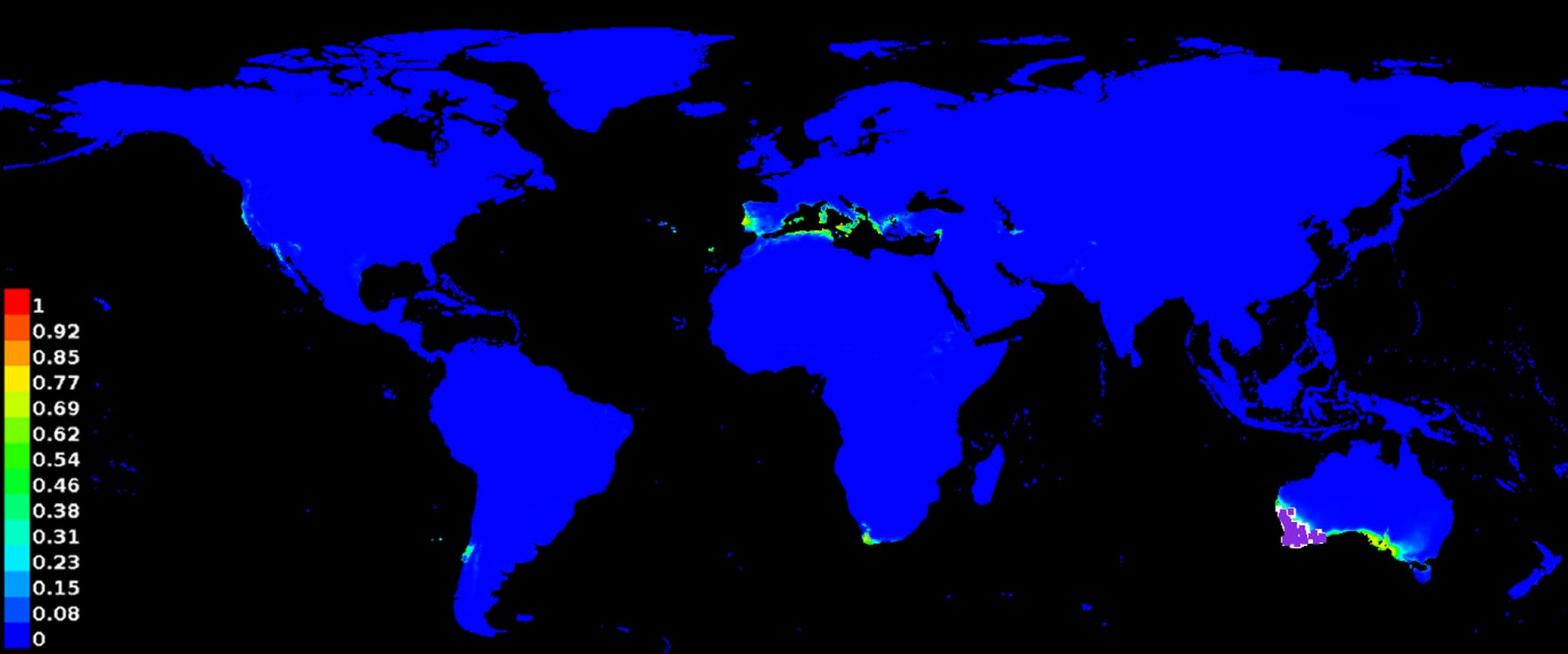
MaxEnt analysis of *A. saligna* produced using the Atlas of Living Australia applying the six BIOCLIM variables used by Thompson et al., (2011). Purple dots indicate natural distribution data from Western Australia used to calibrate the model. The scale at the left of the map indicates climatic suitability with yellow/green areas being relatively suitable and blue unsuitable. Note the extensive climatically suitable areas in the Mediterranean region compared with output from the MaxEnt model shown in Figure 5 of Higgins et al., (2020)

MaxEnt parameter settings and region selection can have important impacts on analysis outputs (Merow et al., 2013), but there is no indication that these could account for the problem described here. All the analyses described here used default parameter settings. The analyses shown here in Figures 1 and 3 were for the whole world. The Thompson et al., (2011) analysis shown in Figure 2 was clipped to include only the Mediterranean region but produced similar results for the region to those shown in Figure 3.

In conclusion, the use of just monthly variables is not appropriate for global application of correlative models. It is recommended that BIOCLIM variables, such as those provided by the WorldClim database (Fick & Hijmans, 2017; Hijmans et al., 2005), should be used. If researchers wish to use other variables derived from monthly values, it is recommended that they compare their detailed SDM results with the broad regions provided by Köppen–Geiger maps (Peel et al., 2007). If there are gross differences, this suggests more variables may be needed or that the variables being used may not be appropriate.

Higgins et al., (2020) is potentially a very interesting paper, but the Köppen–Geiger classification, the Thompson et al., (2011) paper, and the ALA analysis described here clearly indicate that there is a fundamental problem with the MaxEnt analyses as illustrated by Figure 5 in the Higgins et al., (2020) paper. It is hoped that the authors will be able to repeat their analyses using BIOCLIM variables with the MaxEnt model.

## CONFLICT OF INTEREST

None declared.

## AUTHOR CONTRIBUTION

**Trevor Booth:** Conceptualization (lead); Writing‐original draft (lead); Writing‐review & editing (lead).
